# Editorial: Cell and therapeutic delivery using injectable hydrogels for tissue engineering applications

**DOI:** 10.3389/fbioe.2023.1170933

**Published:** 2023-03-22

**Authors:** Ghulam Jalani, Muhammad Rizwan, Muhammad Aftab Akram, Mohammad Mujahid

**Affiliations:** ^1^ Department of Mining and Materials Engineering, McGill University, Montreal, QC, Canada; ^2^ Department of Biomedical Engineering, Michigan Technological University, Houghton, MI, United States; ^3^ Pak-Austria Fachhochschule Institute of Applied Sciences and Technology, Haripur, Pakistan

**Keywords:** biomaterias, hydrogels, tissue engineering, drug delivery, biomedical

Hydrogels, crosslinked networks of hydrophilic polymers, resemble soft living tissues and allow incorporation of cells and therapeutic molecules to stimulate *in-vitro* cellular growth and *in-vivo* regeneration. In particular, the injectable hydrogels, which can be employed to deliver cells and therapeutics to stimulate *in-situ* regeneration, are actively being developed (Dimatteo et al., 2018). The injectable hydrogels are particularly suitable for minimally invasive therapies, where the traditional prefabricated scaffolds require open surgery for transplantation, leading to long healing time and increased patient-care costs (Øvrebø et al., 2022). However, challenges around mechanical integrity, reduced functions of encapsulated cells (Rizwan et al., 2021), biocompatibility, and manufacturing persists and must be overcome before such materials can be deployed in clinical settings at large scale.

This Research Topic focuses on emerging technologies in the development in the field of polymeric injectable hydrogels as carriers for the delivery of cells, and other therapeutics with target applications in tissue engineering and drug delivery. This featured collection includes 5 original research articles which will be of high interest to the researchers in the area of *in-situ* tissue engineering and regenerative medicine. Moreover, two review articles are also part of this Research Topic which provide systemic analysis hydrogel fabrication strategies for clinical translation and vascularization.

*In-vivo* delivery of the immune cells is challenging while maintaining their functions and viability. Cohen et al. developed a new method involving polyethylene glycol-fibrinogen hydrogel microspheres encapsulating alveolar macrophages and epithelial cells for use in respiratory tract model. When exposed to bacterial endotoxin lipopolysaccharide, cells preserved high viability and secreted moderate levels of TNFα, whereas non-encapsulated cells exhibited a burst TNFα secretion and reduced viability. It also had effect on the morphology of cells. The lipopolysaccharide (LPS)-exposed encapsulated macrophages exhibited elongated morphology and out-migration capability from microspheres. This study shows the feasibility of polymer-encapsulated cell delivery to repair pulmonary damage and for general tissue repair.

Injectable, and simultaneously, tissue adhesive hydrogels are exciting hosting materials to carry and deliver, cells, drugs, and biomolecules such as proteins. In this article, Sun et al. present an injectable, adhesive, and self-healing composite hydrogel system loaded with oxybutynin hydrochloride and evaluate its function in the treatment of overactive bladder (OAB). Authors found that the oxybutynin hydrogel could affect the expression of Orail and STIM1 and change the intracellular calcium concentration to improve the progression of the overactive bladder. In this study, authors demonstrated that dextran oxidized by periodate could be used as a macromolecular cross-linking agent of oxybutynin hydrogel. This system regulated Oxybutynin hydrochloride hydrogel mediated Ca2+ entry by increasing the relative expressions of Orail mRNA, STIM1 mRNA, and protein in OAB rats thus improving their therapeutic effect when compared with oxybutynin sustained–release tablets and oxybutynin hydrochloride solution. This exciting research could lay a foundation for improved OAB management and repair.

Chitosan is a common biodegradable, naturally occurring material used to prepare a variety of hydrogels. In this article, Zhou et al. produced lactobionic acid (LA) modified chitosan and cinnamaldehyde (CA) modified chitosan with improved bioactivity and target specificity for reactive oxygen species (ROS)-induced apoptosis of cancer cells. Authors produced nanoparticles from modified chitosan and loaded anticancer drug doxorubicin (DOX). One of the major challenges in DOX delivery is its half-life and associated stability in physiological environment. This study demonstrated that drug-loaded chitosan-LA and chitosan-CA showed improved anti-tumor ability most probably due the fact that DOX’s half-life improved when loaded inside nanoparticles. Target specificity was significantly improved as well due to LA modification. Additionally, nanoparticles are known to exhibit enhanced permeability and retention (EPR) effect which helped improve DOX delivery when compared to free DOX solution.

Heart valves are among the tissues undergoing enormous shear, tensile and flexural stresses due to their dynamic function. Calcified heart valves reduce their dynamic functionality and can lead to heart failures. Current method to treat such conditions involve heart replacement however such replacements involve use of glutaraldehyde as crosslinking agent. Glutaraldehyde is known to have long term residual toxic effects. In this study, Liu et al. explored alternate crosslinking options to simultaneously stabilize three major components to enhance their long-term durability. They use Neomycin Trisulfate, Polyethylene glycol diglycidyl ether and Tannic acid as a substitute for glutaraldehyde, with reduced calcification, degradation, and inflammation. Hybrid crosslinking had multiple benefits including improved hydrophilicity, thermodynamics stability, cytocompatibility, resistance to enzymatic hydrolysis among others in subcutaneous implants in rat. These exciting results are a step forward towards producing highly useful biomaterials with non-toxic crosslinkers which can have far reaching applications in tissue engineering.

Drug delivery and release from hydrogels is governed by a number of factors including polymeric matrix’s chemical and physical structure, porosity, degradability, morphology, pore structure and crosslinking density. Superporous hydrogels are exciting biomaterials for use as drug carriers. Khan et al. reported synthesis and characterization of acrylic acid/hydroxypropyl methylcellulose superporous hydrogels using gas blowing method, crosslinked *via* glycerol. Extensive testing was carried out to evaluate physical properties, *in-vitro* gelling capacity, void fraction examination and *in vivo* analgesic assessment. Furthermore, hydrogels were used to deliver mefenamic acid. Improved mechanical properties, gelling ability, high porosity and low density make these hydrogels potentially exciting material for sustained delivery of small molecular drugs.

In a review article, Xu et al. discuss progress in materials, crosslinkers, and fabrication techniques of hydrogels and provide a cohesive analysis of the design criteria to improve the clinical translation of hydrogel scaffolds. Authors address key design challenges in fabricating microporous scaffolds to combine hydrogels and live cells in a single step with special focus on scale up, sterility and useability in clinical settings. They describe advantages and outstanding challenges involved in commonly used scaffolds fabrication techniques in detail and suggest potential solutions to overcome these obstacles. Moreover, Im et al. summarize the advances in gelatin methacrylate (GelMA) hydrogels for use in tissue engineering and regenerative medicine. These hydrogels have attracted enormous attention due to their ease of fabrication, tunable crosslinking and extracellular matrix-like physical structure, to name a few. Authors focus on applicability of GelMA hydrogels in bioengineering human vascular network *in-vitro* and *in-vivo* and suggest ways to leverage the properties of these hydrogels to improve the vascularization, which is critically needed to improve engineered tissues.

Collectively these research studies provide a snapshot of the exciting progress being made in developing new injectable hydrogel formulations and crosslinking strategies. Future work focusing on improving the viability of encapsulated cells by reducing the mechanical shear during injection (Aguado et al., 2012), and the bioactivity of the encapsulated therapeutics (Ding et al., 2016), could further mature this technology for clinical translation.
